# CXCL8 Drives MMP1 Upregulation and Promotes Metastatic Progression in Oral Cancer Through CXCR1/2-Mediated JAK1/STAT3 Activation

**DOI:** 10.7150/ijbs.115990

**Published:** 2026-02-01

**Authors:** Kuan-Chou Lin, Tsung-Ming Chang, Ying-Sui Sun, Yu-Rou Lin, Chih-Hsin Tang, Ju-Fang Liu

**Affiliations:** 1 School of Dentistry, College of Oral Medicine, Taipei Medical University, Taipei 110, Taiwan.; 2 Department of Oral and Maxillofacial Surgery, Wan Fang Hospital, Taipei Medical University, Taipei 116, Taiwan.; 3 School of Dental Technology, College of Oral Medicine, Taipei Medical University, Taipei City 111, Taiwan.; 4 School of Biomedical Engineering, Taipei Medical University, 250 Wuxing St., Taipei 110, Taiwan.; 5 Department of Pharmacology, School of Medicine, China Medical University, Taichung 404, Taiwan.; 6 Department of Medical Laboratory Science and Biotechnology, College of Medical and Health Science, Asia University, Taichung 413, Taiwan.; 7 Chinese Medicine Research Center, China Medical University, Taichung 404, Taiwan.; 8 Translational Medicine Center, Shin-Kong Wu Ho-Su Memorial Hospital, Taipei City 111, Taiwan.; 9 School of Oral Hygiene, College of Oral Medicine, Taipei Medical University, Taipei City 110, Taiwan.; 10 Department of Medical Research, China Medical University Hospital, China Medical University, Taichung City 404, Taiwan.

**Keywords:** Oral squamous cell carcinoma, CXCL8, Metastasis, MMP1, JAK1/STAT3 pathway

## Abstract

**Background:**

Oral squamous cell carcinoma (OSCC) is an aggressive malignancy, frequently diagnosed at advanced stages with regional and distant metastases that compromise survival. Identifying key molecular regulators of OSCC progression is essential for developing targeted therapies. Although CXCL8 is elevated in OSCC and linked to tumor progression, its precise pro-metastatic mechanisms and downstream effectors remain unclear.

**Methods:**

To identify key regulators of OSCC metastasis, we integrated bioinformatics analysis of multiple GEO datasets and identified CXCL8 as an upregulated hub gene in OSCC tissues. Functional assays were performed in OSCC cell lines (SCC4, SCC9, HSC3) to investigate the role of CXCL8 in cell migration and elucidate its downstream signaling pathways. An orthotopic tongue xenograft mouse model was established to validate the *in vivo* therapeutic relevance of targeting the CXCL8 pathway.

**Results:**

CXCL8 expression was significantly upregulated in OSCC tissues and strongly correlated with enhanced cell motility in OSCC cell lines. High CXCL8 expression was associated with poor overall survival in head and neck cancer patients. Mechanistically, CXCL8 upregulated matrix metalloproteinase 1 (MMP1) expression and enhanced cell migration through activation of the CXCR1/2-JAK1-STAT3 signaling axis. CXCL8 treatment induced JAK1 and STAT3 phosphorylation, promoted STAT3 nuclear translocation, and directly activated MMP1 promoter activity. Pharmacological inhibition or siRNA-mediated silencing of CXCR1/2, JAK1, STAT3, or MMP1 significantly reversed CXCL8-induced cell migration, wound healing, and MMP1 expression. *In vivo*, inhibition of CXCR1/2 reduced CXCL8 and MMP1 expression in primary tumors and cervical lymph nodes, limiting regional metastasis.

**Conclusion:**

This study reveals that CXCL8 drives OSCC metastasis via CXCR1/2-mediated JAK1/STAT3 activation leading to MMP1 transcriptional upregulation. Our findings establish CXCL8 as both a prognostic biomarker and a promising therapeutic target, and suggest that targeting this pathway offers significant therapeutic potential for preventing OSCC metastatic progression.

## Introduction

Oral squamous cell carcinoma (OSCC) represents one of the most prevalent and aggressive malignancies of the head and neck region, characterized by aggressive local invasion and high metastatic potential 1-3. Despite advancements in diagnosis and treatment, including surgery, chemotherapy, and radiotherapy, many OSCC cases are diagnosed at advanced stages with extensive local invasion or nodal involvement 1, 4. These late-stage diagnoses are associated with poor outcomes, reduced quality of life, and limited treatment options. One of the most critical factors influencing prognosis is metastasis, which significantly increases the risk of recurrence and cancer-related mortality 5. Given these challenges, there is an urgent need to identify molecular drivers of OSCC progression and metastasis, which could serve as potential targets for therapeutic intervention. In parallel, clinical studies have shown that OSCC is accompanied by measurable alterations in circulating molecular markers, suggesting that blood-based assays may offer a complementary approach for early detection or monitoring disease progression 6.

The tumor microenvironment plays a pivotal role in cancer progression, with chemokines serving as essential mediators that orchestrate immune cell recruitment, angiogenesis, extracellular matrix remodeling, and malignant cell behavior 7-9. Among the chemokine superfamily, C-X-C motif chemokine ligand 8 (CXCL8), also designated as interleukin-8 (IL-8), has emerged as a critical pro-tumorigenic factor across multiple malignancies, promoting tumor growth, neovascularization, immune evasion, and metastatic spread 10-12. High levels of CXCL8 expression have been documented in multiple malignancies, including lung, liver, breast, and colorectal cancers. CXCL8 predominantly mediates its biological effects through interaction with its receptors, CXC motif chemokine receptors CXCR1 and CXCR2, expressed not only on immune cells but also on epithelial and tumor cells 7, 13. Activation of these receptors can trigger downstream pathways such as Janus kinase (JAK)/ Signal transducer and activator of transcription (STAT), Mitogen-activated protein kinase (MAPK), Phosphoinositide 3-kinase (PI3K)/Akt, and nuclear factor kappa-light-chain-enhancer of activated B cells (NF-κB), all of which are known to promote cell proliferation, survival, migration, and invasion 11, 14. Although elevated CXCL8 levels have been detected in OSCC tissues and saliva 15, the precise molecular mechanisms through which CXCL8 orchestrates OSCC metastasis—particularly the identity of its critical downstream effectors and the signaling pathways governing their expression—remain poorly defined.

To explore this, we aimed to uncover potential molecular targets involved in OSCC metastasis by analyzing gene expression profiles from multiple GEO datasets. Among the top-ranking hub genes, CXCL8 emerged as a prominent candidate due to its consistent upregulation in OSCC specimens and its well-established involvement in inflammation-associated malignancies. However, the specific mechanisms by which CXCL8 drives OSCC metastatic behavior, including its functional downstream targets and the signaling networks mediating their regulation, have not been comprehensively elucidated. This prompted us to systematically investigate the biological role of CXCL8 and dissect the downstream regulatory mechanisms governing OSCC cell migration and invasion. Our study delineates the functional consequences of CXCL8 signaling through the CXCR1/2 receptors and identifies matrix metalloproteinase 1 (MMP1) as a critical downstream effector whose expression is upregulated via activation of the JAK1/STAT3 signaling axis. Through a combination of *in vitro* functional assays and *in vivo* validation, our findings unveil a novel CXCL8-CXCR1/2-JAK1-STAT3-MMP1 regulatory axis that drives OSCC progression and metastasis, thereby establishing this pathway as a promising target for therapeutic intervention in oral cancer.

## Materials and Methods

### Data collection and differential gene expression analysis

To explore gene expression differences in oral squamous cell carcinoma (OSCC), three publicly available microarray datasets—GSE13601, GSE143905, and GSE31056—were obtained from the Gene Expression Omnibus (GEO) database (https://www.ncbi.nlm.nih.gov/geo/) 16-18. GSE13601 includes 29 OSCC and 29 matched normal tissue samples, GSE31056 contains 23 OSCC and 24 normal samples, and GSE143905 comprises 5 OSCC and 5 normal tissue samples. Differentially expressed genes (DEGs) between OSCC and normal tissues were identified using the GEO2R analysis tool, with selection criteria of adjusted p < 0.05 and absolute log2 fold change (|logFC|) > 1.0. Common DEGs across the three datasets were visualized using the Interactive Venn tool (https://www.interactivenn.net/) 19, 20.

### Identification of hub genes

Protein-protein interaction (PPI) networks of DEGs were constructed using the STRING database (https://string-db.org/), with a confidence score threshold of >0.4 21, 22. The resulting PPI network was imported into Cytoscape software for visualization. Key regulatory genes ("hub genes") were identified using the CytoHubba plugin based on degree centrality. The top 10 genes with the highest connectivity scores were considered as hub genes. Further module analysis was conducted using the MCODE plugin to identify densely connected gene clusters 23, 24.

### Reagents and antibodies

Primary antibodies against CXCL8, MMP1, CXCR1, CXCR2, phosphorylated JAK1 (p-JAK1), total JAK1, phosphorylated STAT3 (p-STAT3), total STAT3, and GAPDH, as well as HRP-conjugated anti-rabbit secondary antibodies, were purchased from GeneTex (Irvine, CA, USA). Recombinant human IL-8 (CXCL8) was obtained from PeproTech (Thermo Fisher Scientific, Waltham, MA, USA). Culture inserts for wound healing assays were from ibidi GmbH (Cat. No. 80209). Other chemicals were purchased from Sigma-Aldrich.

### Cell culture

The human OSCC cell lines SCC9, SCC4 (from ATCC) and HSC3 (from Sigma-Aldrich) were used in this study. Cells were maintained in Dulbecco's Modified Eagle Medium (DMEM) supplemented with 10% fetal bovine serum (FBS), 100 U/mL penicillin, and 100 μg/mL streptomycin, and cultured at 37 °C in a humidified incubator with 5% CO₂. To isolate subpopulations with enhanced migratory capacity, SCC4 cells were seeded into the upper chamber of Transwell inserts (8 μm pore size). Cells that migrated to the lower chamber after 24 hours were collected, and repeated selection (10 rounds) generated a highly migratory subpopulation with markedly increased migratory ability.

### Cell migration assay

Transwell migration assays were performed using 24-well inserts with 8 μm pores. Cells (2 × 10⁴) were seeded in the upper chamber in serum-free medium under experimental conditions. After 24 hours, non-migrated cells were removed from the upper surface with a cotton swab. Migrated cells on the lower membrane were fixed in 4% formaldehyde, stained with 0.05% crystal violet, photographed under a microscope, and counted using ImageJ software (version 1.52a, NIH, Bethesda, MD, USA).

### Wound healing assay

For wound healing assays, SCC4 and HSC3 cells (3 × 10⁴) were seeded into two-chamber culture inserts. After 24 hours of incubation, inserts were removed to create a uniform cell-free gap. Cells were then treated with or without CXCL8 and allowed to migrate for 24 hours. Images were captured at 0 and 24 hours using a microscope, and wound closure was analyzed using ImageJ 1.52a.

### Transient transfection

Small interfering RNAs (siRNAs) targeting CXCL8, CXCR1, CXCR2, JAK1, and STAT3, as well as a non-targeting control siRNA, were purchased from Sigma-Aldrich. Transfections were carried out using DharmaFECT 1 reagent (Horizon Discovery, Waterbeach, Cambridge, UK) according to the manufacturer's instructions and incubated for 24 hours before downstream experiments. The siRNA sequences used included: CXCL8: 5'-CUGCGCCAACACAGAAAUU-3', CXCR1: 5'- AUCUGUAAUAUUUGACAUGUC-3', CXCR2: 5'- UCUUACUUAUGGCUUUAUCAU-3', JAK1: 5'-GAAAUGCUGGGAAUUCCAA-3', STAT3: 5'-UGAUUCUUCGUAGAUUGUGCU-3'. Short hairpin (shRNA) targeting the MMP1 plasmid was purchased from the National RNAi Core Facility Platform (RNAi Core, Taipei, Taiwan). The target sequence of the MMP1 shRNA was 5′-TGAAGATGAAAGGTGGACCAA-3′.

### Western blotting analysis

Following treatment, cells were lysed to extract proteins, which were separated using SDS-PAGE and transferred onto PVDF membranes (Merck Millipore, Burlington, MA, USA). Membranes were blocked with 5% nonfat milk in TBST and incubated overnight at 4 °C with primary antibodies (1:1,000 dilution). After washing, membranes were incubated with HRP-conjugated secondary antibodies (1:10,000 dilution) for 1 hour at room temperature. Protein bands were visualized using enhanced chemiluminescence and captured using a UVP imaging system (Analytik Jena, Upland, CA, USA).

### Real-time quantitative polymerase chain reaction (qPCR)

Total RNA was extracted using the easy-BLUE RNA extraction kit (iNtRON Biotechnology, South Korea). First-strand cDNA was synthesized using the qPCRBIO cDNA Synthesis Kit (PCR Biosystems, UK). Quantitative PCR was performed using iTaq Universal SYBR Green Supermix (Bio-Rad, Hercules, CA, USA) on a CFX Connect Real-Time PCR Detection System. All primers were obtained from Sigma-Aldrich. The primer sequences used included: MMP1: Forward: 5'- GTATGCACAGCTTTCCTCCAC-3', Reverse: 5'- TGCCTCCCATCATTCTTCAGG-3', MMP3: Forward: 5'-AGCAAGGACCTCGTTTTCATT-3', Reverse: 5'-GTCAATCCCTGGAAAGTCTTCA-3', MMP10: Forward: 5'- GGGCTCTTTCACTCAGCCAA-3', Reverse: 5'- ATCACACTTGGCTGGCATCT-3', MMP12: Forward: 5'-AGTTTTGATGCTGTCACTAC-3', Reverse: 5'-TTCATAAGCAGCTTCAATGC-3', GAPDH: Forward: 5'-ACAGTGCATGTAGACC-3', Reverse: 5'-TTGAGCACAGGGTACTTA-3'. Gene expression was normalized to GAPDH using the 2^-ΔΔCt^ method.

### Immunofluorescence analysis

To assess STAT3 localization, cells were fixed with 4% formaldehyde for 15 minutes at room temperature and permeabilized with 0.1% Triton X-100 for 10 minutes. After washing, cells were blocked in 3% bovine serum albumin (BSA) prepared in phosphate-buffered saline (PBS) for 1 hour. Cells were then incubated overnight at 4 °C with anti-STAT3 primary antibody (1:200 dilution). The following day, cells were washed and incubated with a FITC-conjugated secondary antibody for 1 hour in the dark. Nuclei were counterstained with DAPI, and fluorescence images were captured using a Nikon ECLIPSE Ti fluorescence microscope equipped with NIS Elements AR software (version 5.02.01).

### Luciferase Reporter Assay

To evaluate STAT3 transcriptional activity, cells (5 × 10⁴ per well) were seeded in 24-well plates and transfected with either a STAT3 promoter-driven or MMP1 promoter-driven luciferase reporter plasmid. The STAT3 reporter plasmid was obtained from Promega (Madison, WI, USA), and the MMP1 reporter construct was purchased from Gene Universal (Chuzhou City, China) 25. Transfections were performed using Lipofectamine 3000 (Thermo Fisher Scientific) according to the manufacturer's protocol. After 24 hours of transfection, cells were treated with recombinant CXCL8 for an additional 24 hours. In some experiments, cells were pretreated with inhibitors targeting CXCR1, CXCR2, JAK1, or STAT3 before CXCL8 stimulation. Cells were then lysed using reporter lysis buffer (E153A, Promega), and luciferase activity was measured using a VICTOR X2 microplate luminometer (PerkinElmer, Waltham, MA, USA).

### *In vivo* orthotopic-xenografted tumor model

To evaluate the *in vivo* relevance of the CXCL8 signaling axis, an orthotopic tongue cancer xenograft model was established in NOD SCID mice (four-week-old males; BioLASCO, Taipei, Taiwan). Mice were maintained under specific pathogen-free conditions and randomly assigned into control and treatment groups. After one week of acclimatization, mice were anesthetized with isoflurane and injected with 2 × 10⁵ HSC3 cells in 25 μL mixture (medium: Matrigel, 1:1) into the lateral tongue using a syringe. Starting from day 7 post-inoculation, mice received intraperitoneal injections of either vehicle or Reparixin (30 mg/kg, every other day), a selective CXCR1/2 inhibitor. On day 21, mice were sacrificed, and lymph nodes, tongues, and blood samples were harvested for further histological and molecular analyses. All procedures were approved by the Institutional Animal Care and Use Committee (IACUC) of Shin Kong Wu Ho-Su Memorial Hospital (Approval No. 114NSTCIACUC010).

### Immunohistochemistry

Paraffin-embedded tumor tissue sections (3 μm thick) were deparaffinized and rehydrated in graded alcohols and distilled water. Antigen retrieval was performed by heating in 10 mM sodium citrate buffer (pH 6.0) at 95-100 °C for 20 minutes. Endogenous peroxidase activity was blocked using peroxidase blocking reagent (Novolink Polymer Detection System, Leica Biosystems, IL, USA). After blocking nonspecific binding, sections were incubated overnight at 4 °C with primary antibodies against Ki-67, CXCL8, and MMP1 (Genetex, USA), followed by incubation with the Novolink polymer secondary antibody for 1 hour at room temperature. Immunoreactivity was visualized with 3,3'-diaminobenzidine (DAB) and counterstained with hematoxylin. Stained sections were evaluated using a light microscope. Quantification of immunostaining was performed based on the percentage of positively stained cells (0-100%) and staining intensity (0 to 3+), generating a composite IHC score (range: 0-300).

### Statistical analysis

Data are presented as mean ± standard deviation (SD). Statistical comparisons between two groups were performed using the unpaired t-test, while comparisons among multiple groups were analyzed by one-way ANOVA. A *p*-value less than 0.05 was considered statistically significant. Analyses were conducted using GraphPad Prism software (version 8.0.2).

## Results

### CXCL8 is overexpressed in oral cancer and correlates with poor survival

Although CXCL8 overexpression in OSCC has been previously reported 15, the precise mechanisms through which it drives tumor progression—particularly metastatic dissemination—remain inadequately characterized. To address this gap, we performed comprehensive re-analysis of multiple independent GEO datasets to confirm the consistent upregulation of CXCL8 in OSCC and to systematically investigate its association with clinical prognosis and functional significance.

**Figure 1:**
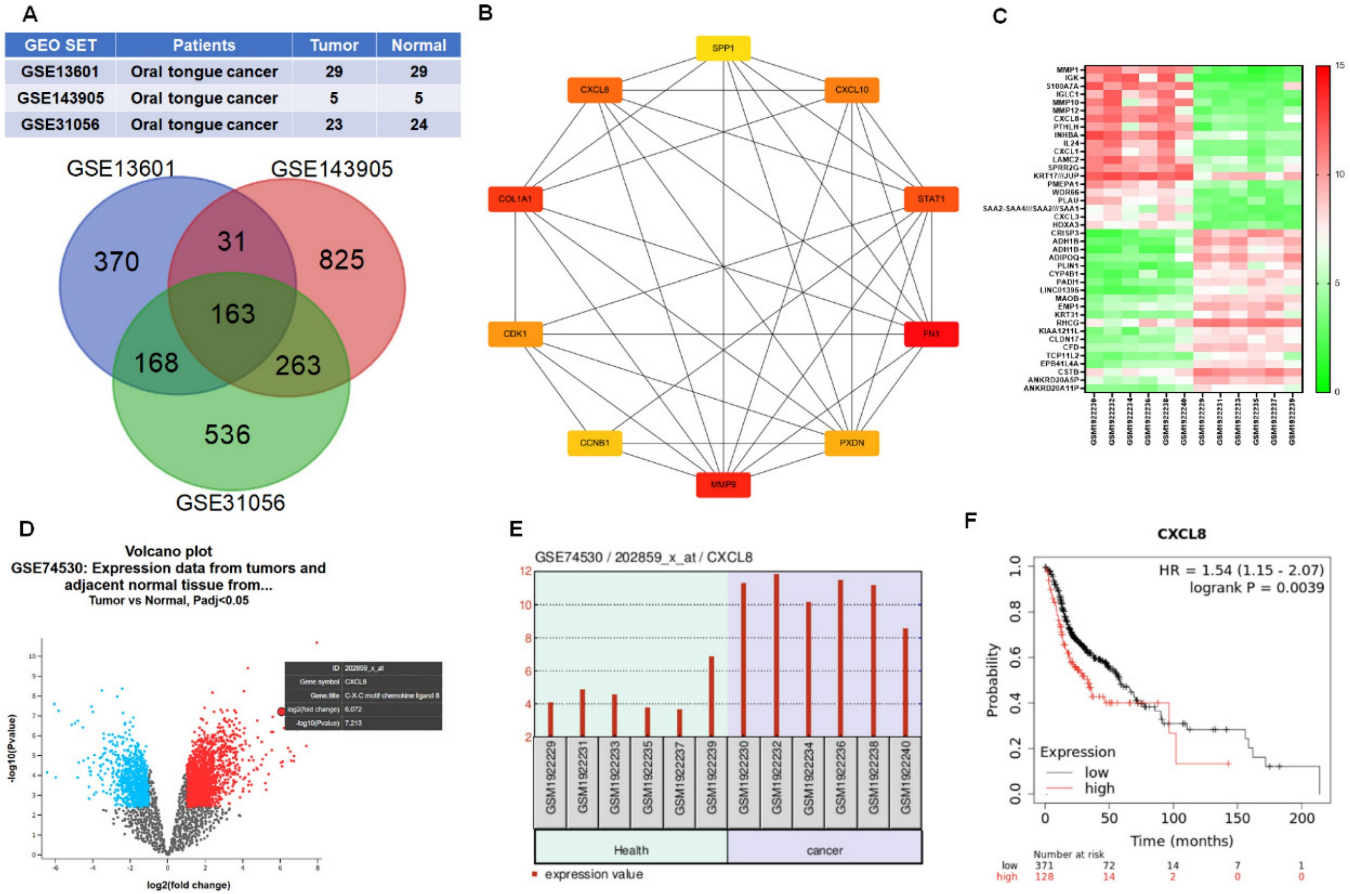
** CXCL8 is overexpressed in OSCC and associated with poor prognosis.** (A) Venn diagram of upregulated DEGs identified from three GEO datasets: GSE13601 (n=29 OSCC, 29 normal), GSE143905 (n=5 OSCC, 5 normal), and GSE31056 (n=23 OSCC, 24 normal). (B) Hub genes ranked by interaction degree within the protein-protein interaction network (Cytoscape). (C) Heatmap showing expression levels of the top 40 upregulated DEGs in OSCC tissues from GSE74530 (n=6 OSCC, 6 normal). (D) Volcano plot of significantly altered genes in GSE74530; red: upregulated, blue: downregulated. (E) Differential expression of CXCL8 between OSCC and normal tissues in GSE74530. (F) Kaplan-Meier analysis showing overall survival in HNSCC patients with high and low CXCL8 expression (UCSC Xena).

To identify key genes associated with oral cancer, we analyzed three publicly available gene expression datasets from the GEO database: GSE13601 (29 OSCC tongue cancer and 29 normal tissues), GSE143905 (5 tongue cancer and 5 normal tissues), and GSE31056 (23 tongue cancer and 24 normal tissues). Differentially expressed genes (DEGs) were identified by comparing tumor and normal samples, focusing on those with significantly increased expression in tongue cancer. A Venn diagram was used to identify overlapping DEGs across all three datasets, revealing 163 genes consistently upregulated in oral tongue cancer (Figure 1A). To explore the potential biological significance of these genes, we constructed a protein-protein interaction (PPI) network using STRING and visualized the network using Cytoscape software. Genes were ranked based on their connectivity within the network, and the top 10 hub genes were identified: CXCL8, SPP1, CXCL10, STAT1, FN1, PXDN, MMP9, CCNB1, CDK1, and COL1A1 (Figure 1B). Notably, these hub genes are functionally enriched in critical cancer-associated processes, including inflammatory signaling, cell cycle regulation, extracellular matrix remodeling, and tumor progression, underscoring their potential relevance in OSCC pathogenesis.

To further validate these findings, we examined the expression profiles of the top 40 upregulated DEGs using another independent GEO dataset (GSE74530), which includes 6 OSCC and 6 normal oral mucosa samples. A heatmap analysis showed a consistent increase in expression of cancer-associated genes in OSCC tissues (Figure 1C). Notably, CXCL8 stood out as one of the most significantly upregulated genes, as confirmed by volcano plot visualization and direct comparison of CXCL8 expression between tumor and normal tissues (Figure 1D-E). Given the established link between inflammation and cancer progression, we next investigated the clinical relevance of CXCL8 in patient outcomes. Using survival data from the UCSC Xena browser, we found that high CXCL8 expression was associated with significantly shorter overall survival in patients with head and neck squamous cell carcinoma (hazard ratio = 1.54), supporting its potential role as a prognostic marker (Figure 1F). Together, these results suggest that CXCL8 is not only a consistently upregulated gene in oral cancer but also may contribute to tumor progression and poor prognosis, highlighting its potential as both a biomarker and a therapeutic target in OSCC.

### CXCL8 expression correlates with cell motility and enhances migration in oral cancer cells

**Figure 2:**
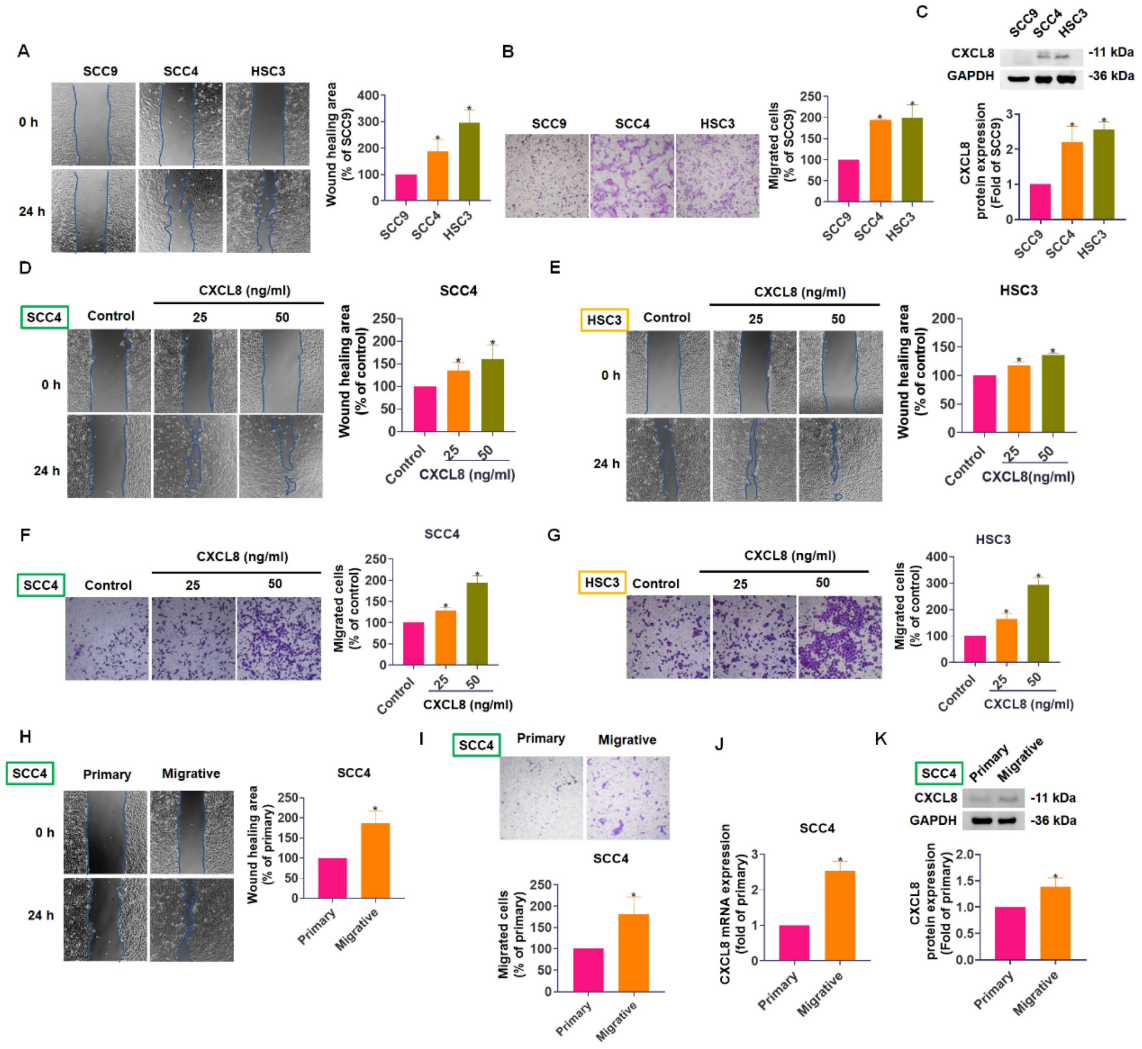
** CXCL8 expression positively correlates with OSCC cell motility.** (A)-(B) Wound healing and Transwell migration assays comparing SCC9, SCC4, and HSC3 cell lines (n=4). (C) Western blot analysis of CXCL8 protein levels in the three cell lines (n=4). (D)-(G) The migration and wound closure were evaluated following CXCL8 (25-50 ng/ml) treatment in SCC4 and HSC3 cells (n=4). (H)-(I) Migration and wound healing ability of SCC4 subpopulations vs. parental SCC4 cells were assessed (n=4). (J)-(K) CXCL8 mRNA and protein expression in highly migratory SCC4 cells compared to parental cells were assessed (n=4). Results are expressed as the mean ± SD of four independent experiments. * *p* < 0.05 compared with the control group.

To further investigate the role of CXCL8 in oral cancer metastasis, we compared the migratory abilities and CXCL8 expression levels of three OSCC cell lines: SCC9, SCC4, and HSC3. In both Transwell migration and wound healing assays, SCC4 and HSC3 cells exhibited significantly greater motility compared to SCC9 cells (Figure 2A-B). Notably, Western blot analysis revealed higher CXCL8 protein expression in SCC4 and HSC3 cells, correlating with their enhanced mobility (Figure 2C). These results suggest a potential link between elevated CXCL8 expression and increased metastatic capacity in OSCC cells.

To directly establish a causal relationship between CXCL8 and enhanced cell motility, we treated SCC4 and HSC3 cells with recombinant human CXCL8 (25-50 ng/mL) and assessed migratory behavior. Exogenous CXCL8 treatment significantly accelerated wound closure in scratch assays (Figure 2D-E) and markedly increased the number of migrated cells in Transwell assays (Figure 2F-G), demonstrating that CXCL8 directly promotes the motile and invasive behavior of OSCC cells in a dose-dependent manner. To further validate this association between CXCL8 expression and metastatic phenotype, we generated a highly migratory subpopulation from parental SCC4 cells through repeated rounds of Transwell-based selection. These selected cells (referred to as highly mobile cells) displayed markedly enhanced migration and wound healing abilities compared to the parental population (Figure 2H-I). Analysis of CXCL8 levels in these highly mobile cells showed a substantial increase in both mRNA and protein expression compared to primary SCC4 cells (Figure 2J-K). Together, these findings suggest that CXCL8 not only correlates with cell motility but actively contributes to the metastatic potential of OSCC cells. This highlights CXCL8 as a promising molecular driver of oral cancer progression and a potential therapeutic target for limiting metastasis.

### CXCL8 promotes oral cancer cell metastasis by increasing MMP1 expression

**Figure 3:**
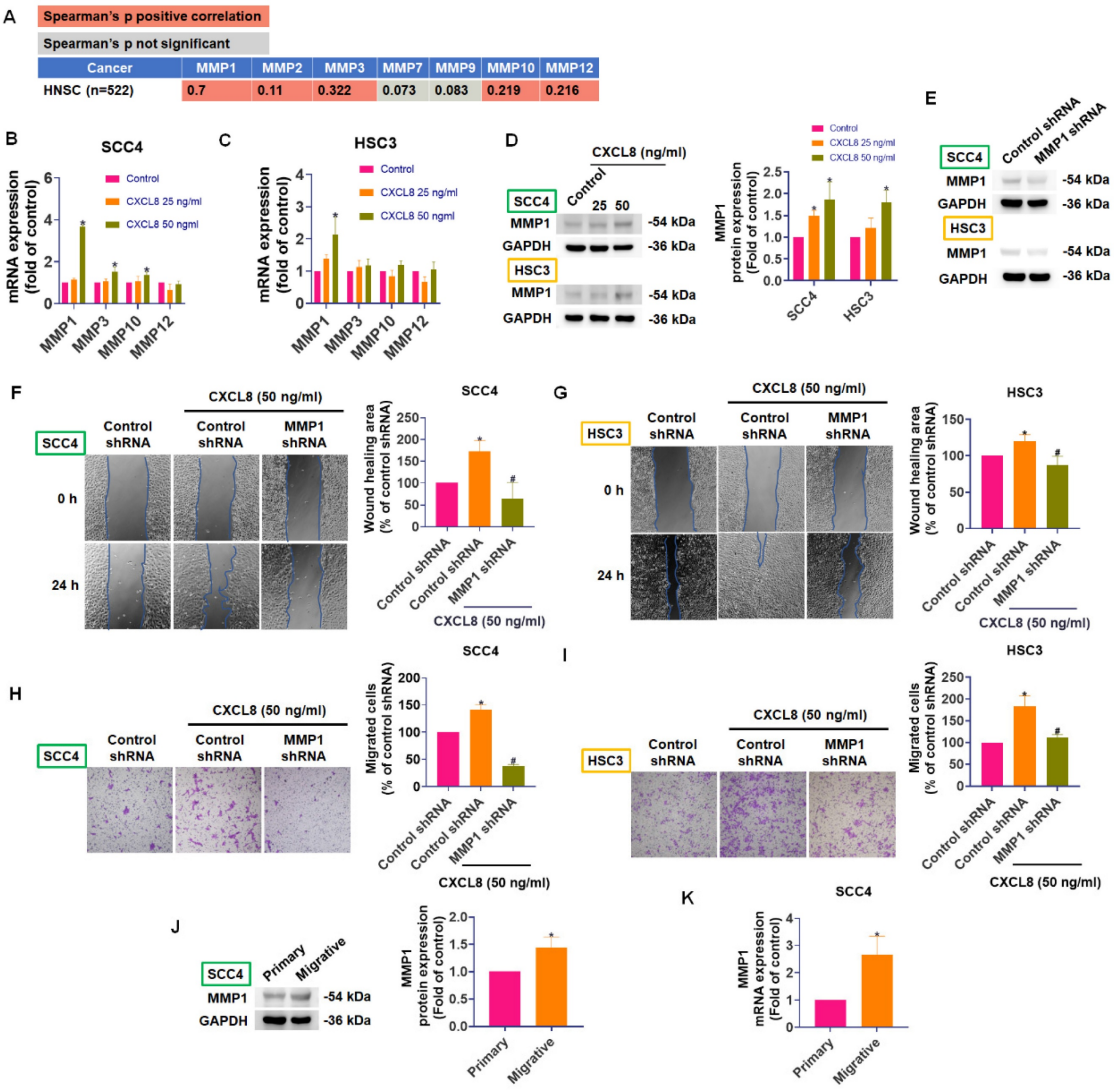
** CXCL8 promotes OSCC cell migration by inducing MMP1 expression.** (A) TIMER2.0 analysis of correlations between CXCL8 and MMPs in HNSC (n=522). (B)-(D) CXCL8-induced MMP1 expression was examined by qPCR and Western blot analysis in SCC4 and HSC3 cells (n=4). (E) Validation of MMP1 knockdown efficiency by Western blot (n=4). (F)-(I) Functional analysis confirmed the effect of MMP1 knockdown on CXCL8-induced cell migration and wound healing (n=4). (J)-(K) MMP1 mRNA and protein expression of highly migratory SCC4 subpopulations were analyzed by Western blotting and qPCR (n=4). Results are expressed as the mean ± SD of four independent experiments. * *p* < 0.05 compared with the control group; # *p* < 0.05 compared with the CXCL8-treated group.

Matrix metalloproteinases (MMPs) are enzymes critical for tumor metastasis due to their ability to degrade extracellular matrix (ECM) components, facilitating cancer cell migration, invasion, and eventual spread to distant sites 26, 27. In cancer progression, both tumor cells and surrounding stromal cells cooperate to produce MMPs, particularly MMP-1, MMP-9, and MMP-13, whose elevated levels often correlate with advanced tumor stage and poor prognosis in oral and other cancers 28-30. To determine which MMP family members might be regulated by CXCL8 in oral cancer, we used TIMER2.0 to analyze correlations between CXCL8 and MMP expression in head and neck cancers (n=522). CXCL8 expression was positively correlated with several MMPs, especially MMP1 (correlation coefficient = 0.7), indicating a potential regulatory relationship (Figure 3A). Next, we evaluated whether CXCL8 directly influences MMP expression in OSCC cells. Quantitative PCR analyses showed that treating SCC4 and HSC3 cells with CXCL8 (50 ng/ml) significantly enhanced MMP1 mRNA expression (Figure 3B-C). Western blot analysis further confirmed a marked increase in MMP1 protein levels following CXCL8 (25-50 ng/ml) stimulation (Figure 3D). To validate the functional role of MMP1 in CXCL8-induced metastasis, we knocked down MMP1 using siRNA and examined cell migration. Transwell migration and wound healing assays demonstrated that silencing MMP1 significantly reduced CXCL8-induced cell motility, confirming that MMP1 is essential for the metastatic effects mediated by CXCL8 (Figure 3E-I). Moreover, SCC4 cells selected for higher migratory capacity consistently expressed higher levels of MMP1 at both the protein and mRNA levels (Figure 3J-K). Collectively, these results confirm a strong positive correlation between CXCL8 and MMP1 and establish that CXCL8 facilitates oral cancer cell metastasis by specifically upregulating MMP1 expression.

### CXCL8 enhances oral cancer cell metastasis by upregulating MMP1 through CXCR1 and CXCR2 receptors

**Figure 4:**
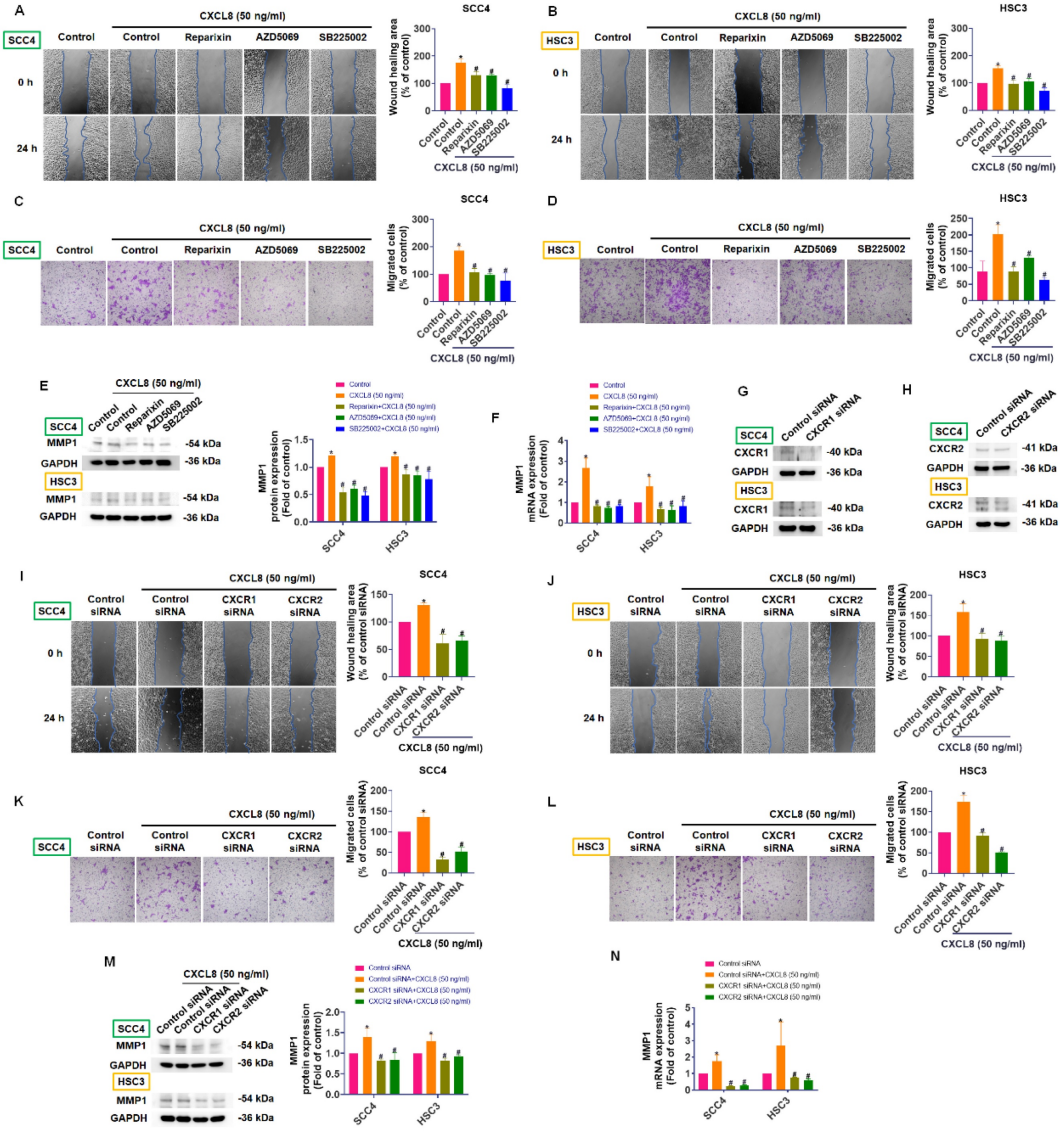
** CXCL8 enhances MMP1 expression and migration via CXCR1/2 receptors.** (A)-(D) Wound healing and cell migration abilities were evaluated by pre-treating CXCR1/2 inhibitors (Reparixin: 1 nM, AZD5069: 1 μM and SB225002: 200 nM) before CXCL8 (50 ng/ml) stimulation (n=4). (E)-(F) MMP1 expression was evaluated by pre-treating CXCR1/2 inhibitors (Reparixin: 1 nM, AZD5069: 1 μM and SB225002: 200 nM) before CXCL8 stimulation (n=4). (G)-(H) Validation of CXCR1 and CXCR2 siRNA knockdown efficiency (n=4). (I)-(L) Migration and wound healing assays were used to confirm the effect of CXCR1/2 knockdown on CXCL8-induced cell mobility (n=4). (M)-(N) MMP1 expression was evaluated at both mRNA and protein levels after CXCR1/2 knockdown and CXCL8 stimulation (n=4). Results are expressed as the mean ± SD of four independent experiments. * *p* < 0.05 compared with the control group; # *p* < 0.05 compared with the CXCL8-treated group.

CXCL8 exerts its biological effects, including promoting cancer cell migration and invasion, primarily through binding to its specific receptors CXCR1 and CXCR2, thereby activating downstream signaling pathways 7, 11, 13, 14. To verify whether these receptors mediate CXCL8-induced metastasis in oral cancer cells, we utilized selective inhibitors targeting CXCR1 and CXCR2 (Reparixin, AZD5069, and SB225002). Pre-treatment with these inhibitors markedly reduced CXCL8-induced cell migration and wound-healing capability in SCC4 and HSC3 cells (Figure 4A-D). Consistently, inhibition of CXCR1 and CXCR2 significantly suppressed the CXCL8-mediated increase in MMP1 mRNA and protein expression (Figure 4E-F). To further confirm these findings, we employed siRNA-mediated knockdown of CXCR1 and CXCR2 receptors. Efficient silencing of CXCR1 and CXCR2 notably reversed the enhanced migration and wound healing responses triggered by CXCL8 (Figure 4G-L). Correspondingly, knockdown of CXCR1 and CXCR2 substantially inhibited the CXCL8-driven upregulation of MMP1 at both the protein and mRNA levels (Figure 4M-N). These data collectively indicate that CXCR1 and CXCR2 receptors are essential for CXCL8-induced MMP1 expression and subsequent metastatic process in oral cancer cells. Thus, targeting the CXCL8-CXCR1/2-MMP1 signaling axis represents a promising approach to inhibit OSCC metastasis.

### CXCL8 promotes oral cancer metastasis by activating MMP1 expression through the CXCR1/2-JAK1/STAT3 signaling pathway

**Figure 5:**
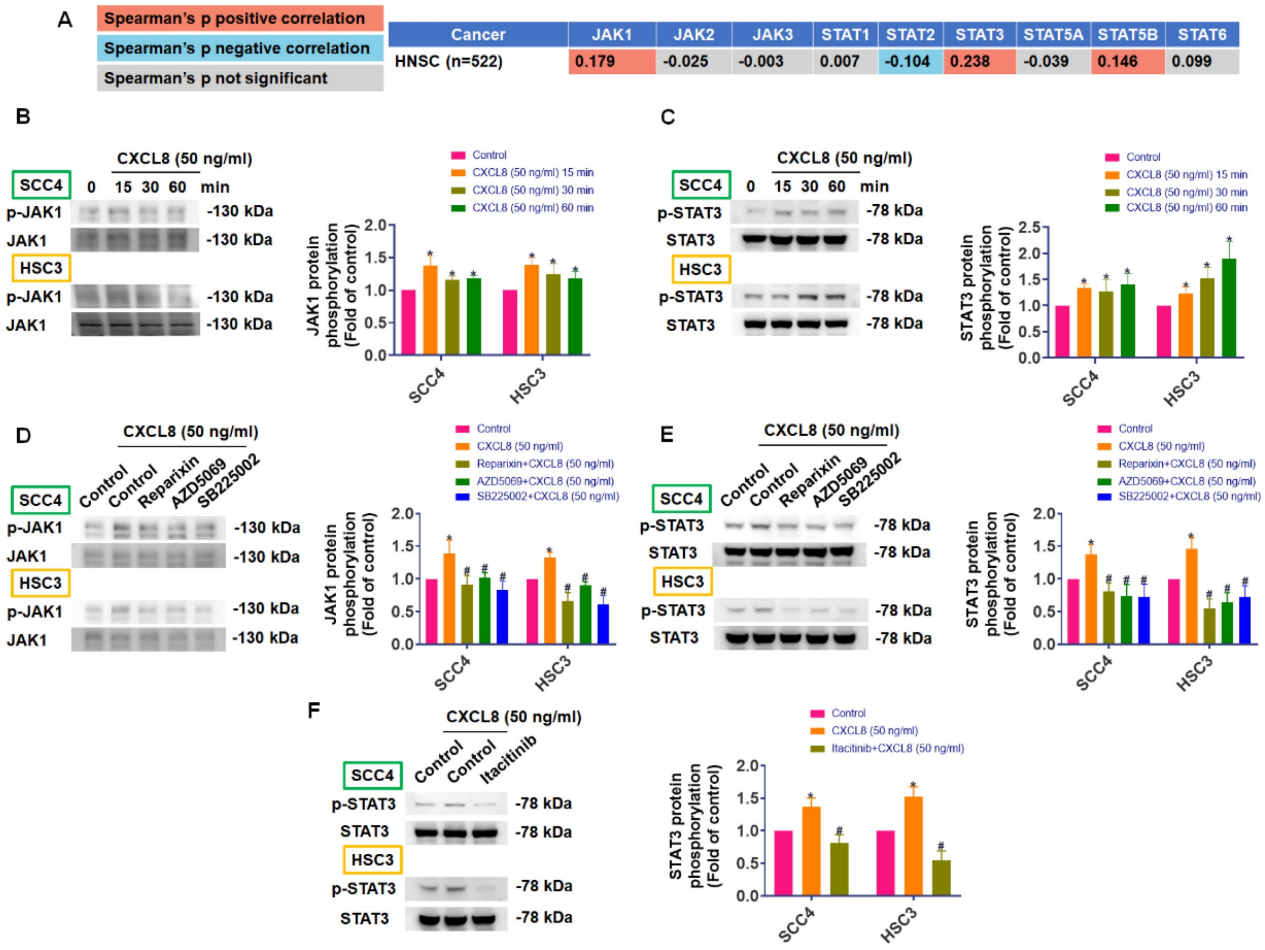
** CXCL8 activates JAK1/STAT3 signaling via CXCR1/2.** (A) TIMER2.0 analysis showing correlation between CXCL8 and JAK/STAT pathway genes in HNSC. (B)-(C) CXCL8-induced phosphorylation of JAK1 and STAT3 in SCC4 and HSC3 was assessed using Western blot analysis (n=4). (D)-(E) Cells receiving pretreatment with CXCR1/2 inhibitors and subsequent CXCL8 incubation, phosphorylation of JAK1 and STAT3 was assessed using Western blot analysis (n=4). (F) JAK1 inhibition (Itacitinib: 50 nM) was followed by CXCL8 stimulation, and STAT3 phosphorylation was assessed by Western blotting (n=4). Results are expressed as the mean ± SD of four independent experiments. * *p* < 0.05 compared with the control group; # *p* < 0.05 compared with the CXCL8-treated group.

**Figure 6:**
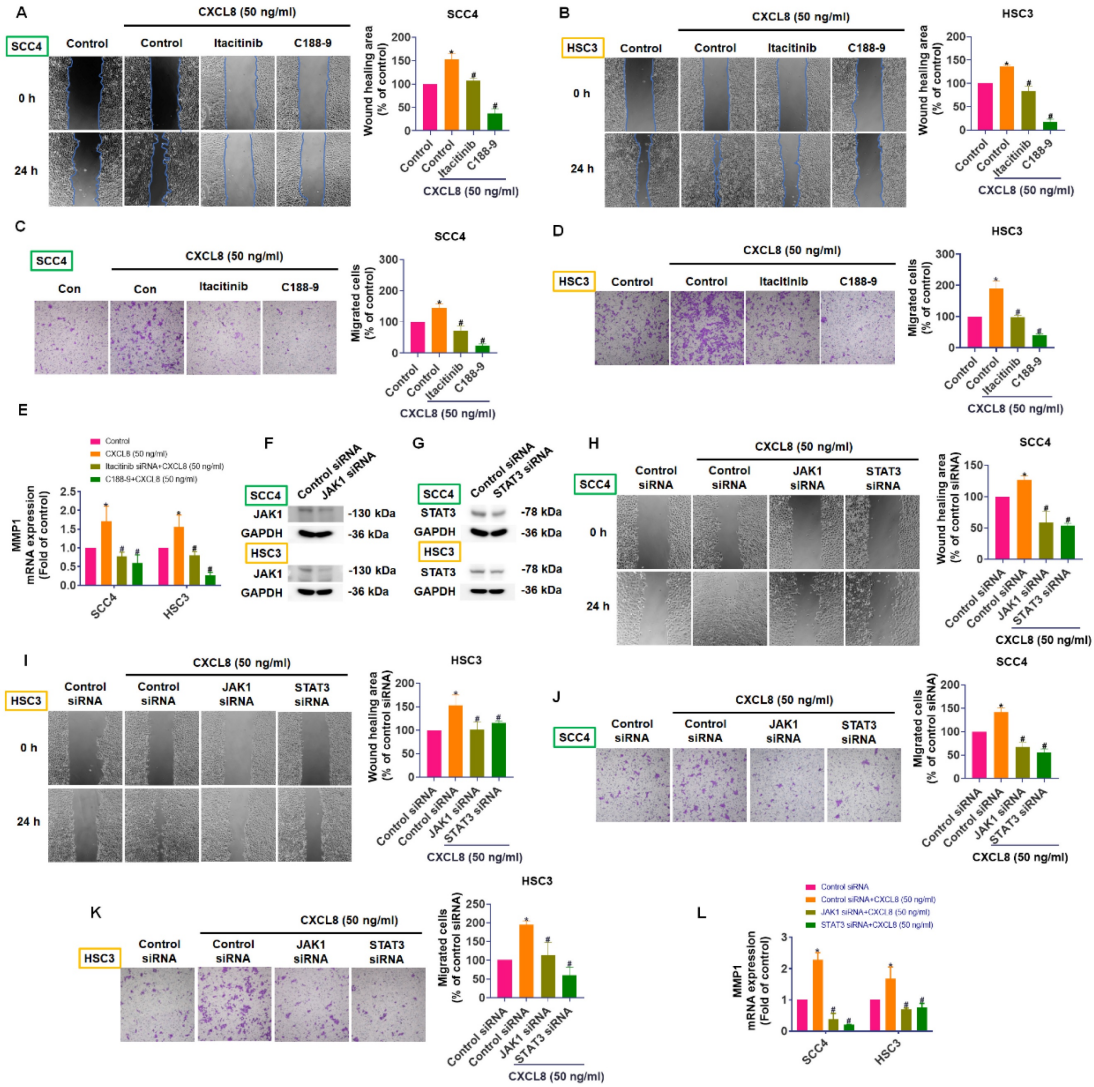
** JAK1/STAT3 signaling is essential for CXCL8-mediated migration and MMP1 expression.** (A)-(D) CXCL8-induced migration and wound healing were assessed after incubation with JAK1 (Itacitinib) and STAT3 (C188-9: 5 μM) inhibitors (n=4). (E) CXCL8-induced MMP1 mRNA was assessed using qPCR after incubation with JAK1 (Itacitinib) and STAT3 (C188-9) inhibitors (n=4). (F)-(G) Confirmation of JAK1 and STAT3 knockdown by Western blot (n=4). (H)-(K) CXCL8-mediated cell migration and wound healing were evaluated following siRNA knockdown of JAK1/STAT3 (n=4). (L) CXCL8-induced MMP1 mRNA expression was assessed following JAK1/STAT3 knockdown (n=4). Results are expressed as the mean ± SD of four independent experiments. * *p* < 0.05 compared with the control group; # *p* < 0.05 compared with the CXCL8-treated group.

CXCL8 can activate several downstream signaling pathways—including JAK/STAT, MAPK, PI3K/Akt, and NF-κB—to enhance cancer progression and metastasis, although the exact mechanisms in OSCC remain incompletely understood 11, 14. Using TIMER2.0 database analysis, we identified positive correlations between CXCL8 and components of the JAK/STAT pathway in head and neck cancers, specifically with JAK1 (0.179) and STAT3 (0.238), and a negative correlation with STAT2 (-0.104), suggesting a potential role for CXCL8 in activating the JAK1/STAT3 axis (Figure 5A). To confirm this experimentally, we assessed phosphorylation levels of JAK1 and STAT3 in SCC4 and HSC3 cells after treatment with recombinant CXCL8 (50 ng/ml). Western blot results demonstrated that CXCL8 treatment significantly enhanced phosphorylation of both JAK1 and STAT3 proteins, indicating activation of this signaling pathway (Figure 5B-C). To further validate that this activation occurs through the CXCL8 receptors CXCR1 and CXCR2, we pre-treated cells with CXCR1/2 inhibitors (Reparixin, AZD5069, and SB225002). This pre-treatment markedly inhibited CXCL8-induced phosphorylation of JAK1 and STAT3, confirming receptor-mediated activation (Figure 5D-E). Similarly, inhibition of JAK1 using the selective inhibitor Itacitinib significantly blocked STAT3 phosphorylation induced by CXCL8, verifying that JAK1 acts upstream of STAT3 activation (Figure 5F). These results clearly show that CXCL8 signals through CXCR1/2 to activate the JAK1/STAT3 pathway in oral cancer cells. Next, we performed functional assays to determine if the JAK1/STAT3 pathway mediates CXCL8-driven OSCC metastasis. Both the JAK1 inhibitor (Itacitinib) and the STAT3 inhibitor (C188-9) significantly reduced CXCL8-induced cell migration and wound healing (Figure 6A-D). Consistent with these functional effects, inhibition of JAK1 or STAT3 also markedly suppressed CXCL8-induced MMP1 mRNA expression (Figure 6E). We further validated these results by knocking down JAK1 and STAT3 using siRNA. Effective silencing of JAK1 and STAT3 significantly reversed the enhancement of cell migration and wound healing induced by CXCL8 (Figure 6F-K). Likewise, siRNA-mediated reduction of JAK1 or STAT3 also significantly decreased CXCL8-stimulated MMP1 expression (Figure 6L). Together, these findings confirm a clear signaling cascade wherein CXCL8 binds to CXCR1/2, triggering JAK1 and subsequent STAT3 activation, resulting in increased MMP1 expression and promoting OSCC metastasis. These insights provide a valuable foundation for targeting the CXCL8-CXCR1/2-JAK1-STAT3 axis as a therapeutic strategy against oral cancer progression.

### STAT3 directly mediates CXCL8-induced MMP1 transcription

**Figure 7:**
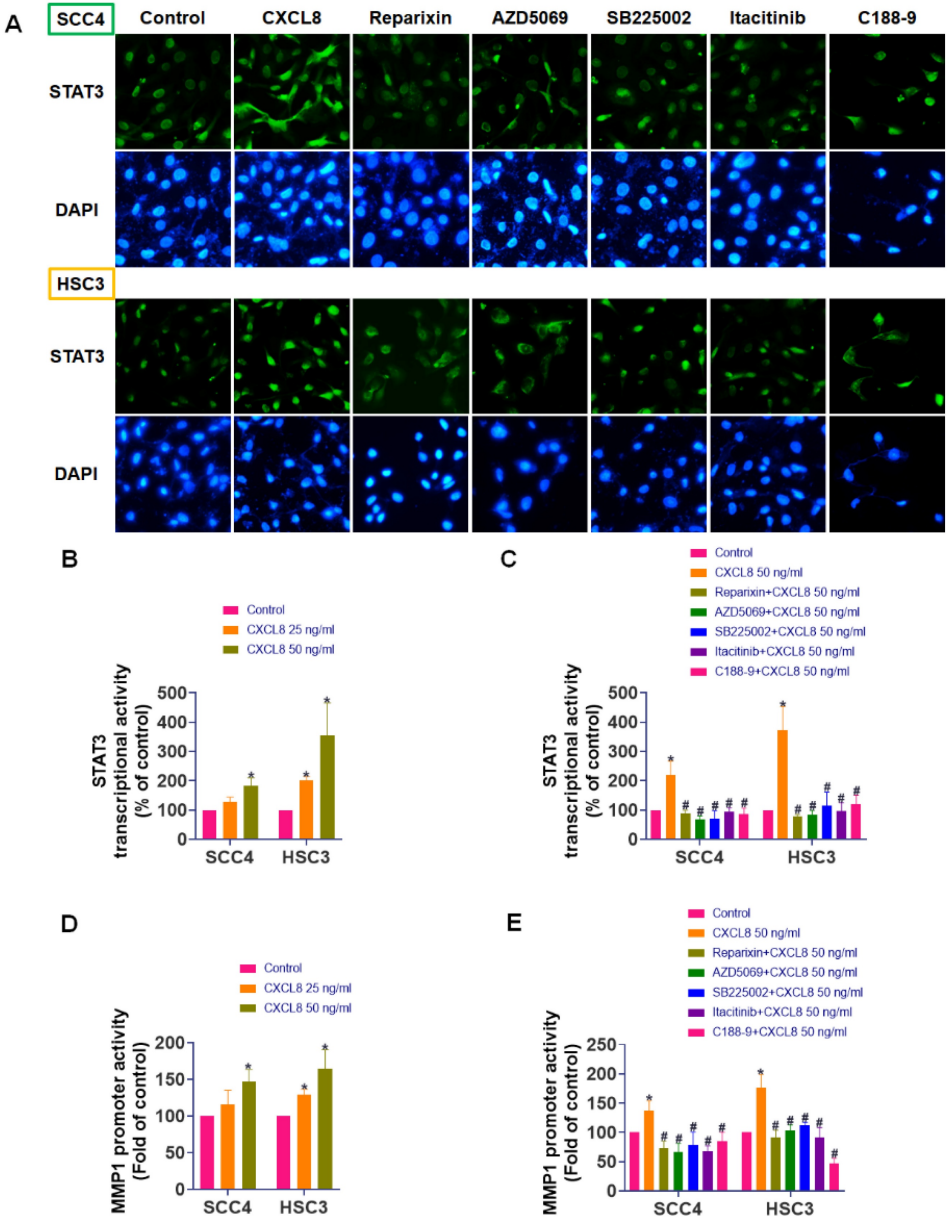
** CXCL8 promotes STAT3 nuclear translocation and MMP1 promoter activation via CXCR1/2/JAK1 signaling.** (A) After pretreatment with CXCR1/2/JAK1/STAT3 inhibitors, immunofluorescence was used to observe CXCL8-induced STAT3 nuclear translocation. (B) Luciferase reporter assay evaluating STAT3 promoter activity in SCC4 and HSC3 cells treated with CXCL8 for 24 hours (n=4). (C) Luciferase reporter assay performed as in (B), with additional pretreatment using inhibitors targeting CXCR1/2, JAK1, or STAT3 (n=4). (D) Luciferase reporter assay evaluating MMP1 promoter activity in SCC4 and HSC3 cells treated with CXCL8 for 24 hours (n=4). (E) Luciferase reporter assay performed as in (D), with additional pretreatment using inhibitors targeting CXCR1/2, JAK1, or STAT3 (n=4). Results are expressed as the mean ± SD of four independent experiments. * *p* < 0.05 compared with the control group; # *p* < 0.05 compared with the CXCL8-treated group.

To fully elucidate how CXCL8 activates downstream signaling pathways to promote oral cancer metastasis, we explored its effect on STAT3 nuclear translocation and transcriptional activity. SCC4 and HSC3 cells were treated with CXCL8 for 2 hours following pre-incubation with selective inhibitors targeting CXCR1/2, JAK1, or STAT3. Immunofluorescence staining revealed that CXCL8 induced substantial nuclear localization of STAT3, indicating its activation. In contrast, treatment with inhibitors targeting CXCR1/2, JAK1, or STAT3 resulted in a marked reduction of STAT3 nuclear accumulation, with signals predominantly observed in the cytoplasm (Figure 7A). To further validate CXCL8-induced transcriptional activation of STAT3, we performed luciferase reporter assays. CXCL8 (50 ng/ml) significantly increased STAT3 transcriptional activity, an effect that was markedly reversed by CXCR1/2, JAK1, or STAT3 inhibitors (Figure 7B-C). These findings strongly indicate that CXCL8 promotes STAT3 nuclear translocation and subsequent transcriptional regulation specifically through the CXCR1/2-JAK1 signaling pathway. In addition, to determine whether this signaling axis drives the expression of metastasis-related genes, we performed a luciferase assay using an MMP1 promoter reporter construct. CXCL8 significantly increased MMP1 promoter activity (Figure 7D). Consistent with inhibition of upstream pathways, pretreatment with CXCR1/2, JAK1, or STAT3 inhibitors significantly inhibited CXCL8-induced MMP1 transcriptional activation (Figure 7E). These findings strongly demonstrate that CXCL8 promotes OSCC cells migration by triggering STAT3 nuclear translocation and transcriptional activation, which in turn directly enhances MMP1 gene expression. Collectively, these findings provide comprehensive molecular evidence that CXCL8 drives OSCC metastasis through CXCR1/2-mediated JAK1/STAT3 activation, culminating in transcriptional induction of the pro-metastatic effector MMP1.

### CXCL8 promotes oral cancer metastasis via the CXCR1/2-JAK1-STAT3-MMP1 axis *in vitro* and *in vivo*

**Figure 8:**
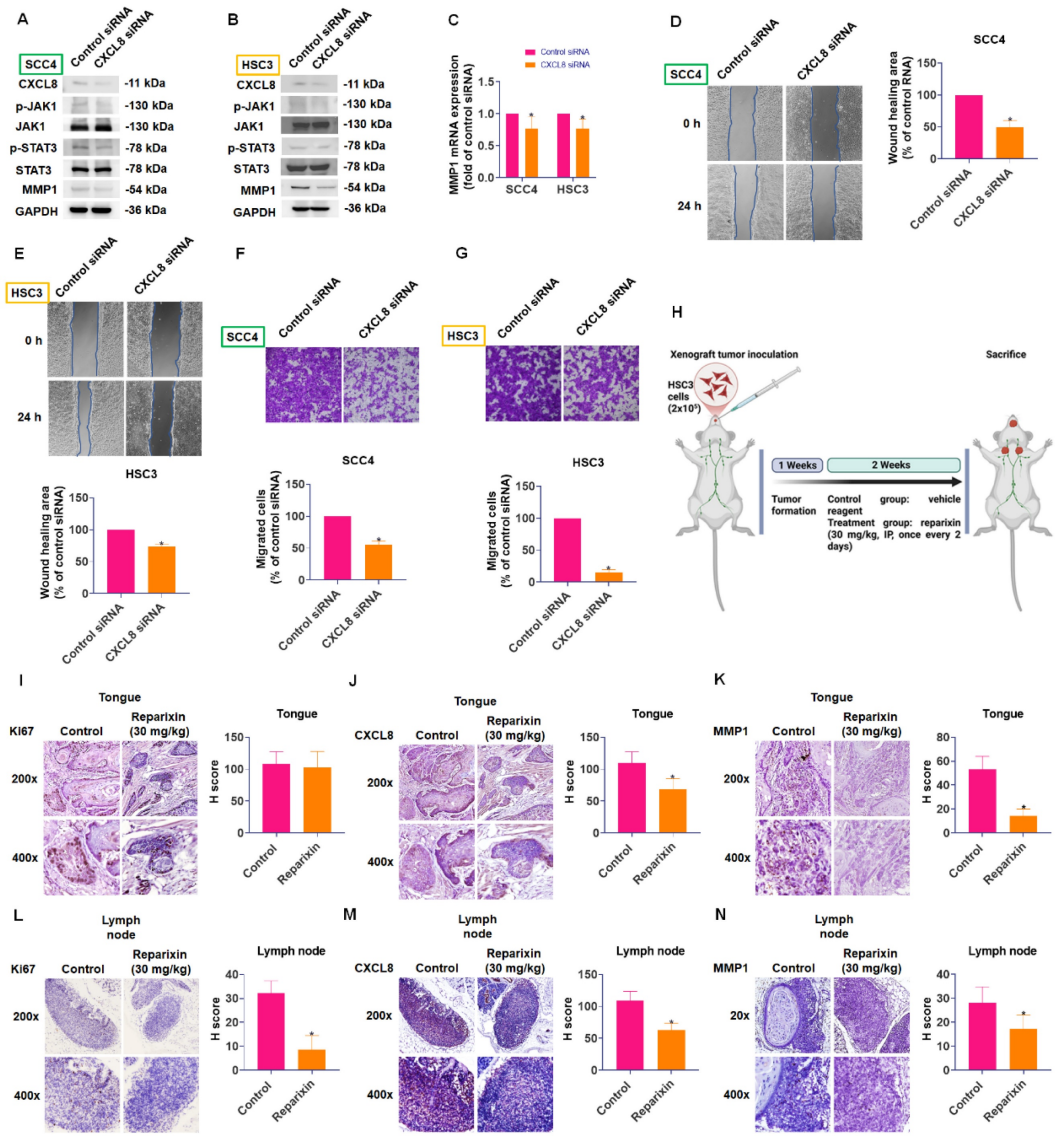
** CXCL8 promotes oral cancer metastasis *in vitro* and *in vivo*.** (A-B) Western blot analysis demonstrated the effect of CXCL8 knockdown on JAK1 and STAT3 phosphorylation, and MMP1 protein levels (n=4). (C) MMP1 mRNA expression was evaluated following CXCL8 knockdown (n=4). (D)-(G) The migration and wound healing ability was assessed following CXCL8 knockdown in SCC4 and HSC3 cells (n=4). (H) Schematic representation of the *in vivo* orthotopic xenograft model using HSC3 cells injected into the lateral tongue of NOD/SCID mice, followed by treatment with vehicle or reparixin (30 mg/kg) every other day for two weeks. (I-K) IHC staining and H-score quantification of Ki67, CXCL8, and MMP1 expression in tongue tumors from control and reparixin-treated mice (n=4). (L-N) IHC staining and H-score quantification of Ki67, CXCL8, and MMP1 expression in cervical lymph node tissues (n=4). Results are expressed as the mean ± SD of four independent experiments. * *p* < 0.05 compared with the control group.

To confirm the functional significance of CXCL8 in oral cancer metastasis, we employed CXCL8-targeted siRNA to reduce its expression. Western blot analysis showed that knocking down CXCL8 resulted in a significant decrease in phosphorylation of both JAK1 and STAT3 proteins. Concurrently, we observed substantial reductions in MMP1 expression at both mRNA and protein levels (Figure 8A-C). Additionally, silencing CXCL8 in highly migratory SCC4 and HSC3 cells significantly suppressed their migration and wound-healing capacities (Figure 8D-G).

To validate the therapeutic relevance of targeting the CXCL8 pathway *in vivo*, we established an orthotopic tongue xenograft model by injecting HSC3 cells into the lateral tongue of NOD/SCID mice (Figure 8H). Immunohistochemical (IHC) analysis of primary tongue tumors revealed no significant difference in Ki67 expression between the vehicle- and reparixin-treated groups (Figure 8I), indicating comparable proliferative activity. However, CXCL8 and MMP1 expression levels were significantly reduced in the reparixin-treated tumors (Figure 8J-K), suggesting effective inhibition of the CXCL8/CXCR1/2 signaling axis. Interestingly, reparixin treatment also led to a marked reduction in CXCL8 expression within the primary tumor tissue, suggesting a potential feedback mechanism. This observation raises the possibility that downstream effectors, such as STAT3, may participate in sustaining CXCL8 expression through a self-reinforcing regulatory loop. Moreover, analysis of cervical lymph nodes showed that reparixin treatment resulted in reduced tumor dissemination, as evidenced by fewer Ki67-positive tumor cells and lower CXCL8 and MMP1 expression levels (Figure 8L-N). To identify the cellular source of CXCL8, we analyzed the GSE103322 single-cell RNA-seq dataset using the UCSC Cell Browser. We found that malignant epithelial cells were the dominant source of CXCL8, which also co-expressed MMP1 (Supplementary Figure 1A-C). These findings suggest that although reparixin did not significantly alter primary tumor proliferation, it effectively suppressed pro-metastatic signaling and reduced regional lymphatic spread.

Taken together, these findings demonstrate that CXCL8 critically promotes oral cancer metastasis through CXCR1/2-dependent activation of the JAK1/STAT3 pathway, ultimately enhancing STAT3 nuclear translocation, transcriptional activation, and MMP1 expression. Clarifying these detailed molecular mechanisms highlights CXCL8 as a potential biomarker and therapeutic target for managing metastatic oral cancer.

## Discussion

The treatment of oral squamous cell carcinoma (OSCC) continues to face significant clinical challenges, primarily due to late diagnosis, extensive local invasion, and metastatic spread, which limit effective therapeutic strategies. Current treatments typically involve surgery combined with chemotherapy or radiotherapy; however, these interventions are often insufficient to control metastasis, a critical factor contributing to poor patient survival 31, 32. The lack of effective molecularly targeted therapies highlights the urgent need for identifying novel targets that can specifically intervene in the metastatic process 5. CXCL8 is a well-recognized pro-inflammatory chemokine, with known overexpression in several cancers, including OSCC. However, the precise molecular mechanism linking CXCL8 to metastatic behavior in OSCC has remained poorly defined. Our study addresses this gap by identifying and validating the CXCL8-CXCR1/2-JAK1-STAT3-MMP1 signaling axis as a key driver of OSCC metastasis. Unlike previous studies that simply documented CXCL8 expression, we provide direct evidence that CXCL8 promotes STAT3 nuclear translocation and transcriptional activation, thereby upregulating MMP1 and enhancing tumor cell migration.

CXCL8 is a well-characterized pro-inflammatory chemokine implicated in multiple physiological and pathological processes, including inflammation, angiogenesis, and tumor progression 33. Elevated CXCL8 expression has been correlated with poor prognosis across various malignancies—such as colorectal, hepatocellular, and breast cancers—owing to its roles in promoting tumor vascularization, invasion, and immune evasion 34-38. These effects are primarily mediated through binding to its cognate receptors, CXCR1 and CXCR2, which activate downstream signaling cascades including JAK/STAT, MAPK, and NF-κB pathways 39-42. In our study, we demonstrate that CXCL8 promotes OSCC metastasis by activating the CXCR1/2-JAK1-STAT3 axis, leading to upregulation of MMP1 and enhanced tumor cell migration. Notably, inhibition of CXCR1/2 or downstream components such as JAK1 and STAT3 significantly suppressed CXCL8-induced signaling and reduced cell motility. These findings not only reveal a novel CXCL8-driven metastatic mechanism in OSCC but also highlight the therapeutic potential of targeting this pathway. Several inhibitors of CXCR1/2 and STAT3 are currently undergoing clinical evaluation in other malignancies 43, 44, underscoring the translational relevance of our results for future OSCC treatment strategies.

Among the MMP family members, MMP1 has been specifically implicated in OSCC progression and metastasis, with studies demonstrating that MMP1 expression is significantly elevated in invasive and metastatic OSCC tissues and correlates with poor clinical outcomes 45. Recent investigations have established that MMP1 is upregulated in head and neck squamous cell carcinoma (HNSCC) tissues and directly enhances the metastatic ability of cancer cells by degrading type I collagen and other ECM components, thereby facilitating tumor cell migration through tissue barriers 46. Immunohistochemical studies have further revealed that MMP1 expression increases progressively from normal oral mucosa to well-differentiated and poorly differentiated OSCC, suggesting that MMP1 upregulation is a key event in oral cancer progression 47. Given that MMP1 overexpression is associated with enhanced invasiveness and metastatic capabilities in OSCC, modulating MMP1 activity represents a viable therapeutic approach to prevent tumor invasion and metastasis 48. Despite the well-established role of MMP1 in OSCC metastasis, the upstream regulatory mechanisms governing its expression have remained incompletely defined. Our study addresses this critical knowledge gap by demonstrating that CXCL8 directly upregulates MMP1 expression through activation of the CXCR1/2-JAK1-STAT3 signaling axis. We provide multiple lines of evidence supporting this regulatory mechanism: (1) CXCL8 treatment dose-dependently increased MMP1 mRNA and protein expression in OSCC cells; (2) pharmacological inhibition or siRNA-mediated knockdown of CXCR1/2, JAK1, or STAT3 abolished CXCL8-induced MMP1 upregulation; (3) CXCL8 stimulation promoted STAT3 nuclear translocation and directly activated the MMP1 promoter, as demonstrated by luciferase reporter assays; and (4) functional inhibition of MMP1 significantly attenuated CXCL8-induced cell migration and invasion.

To validate these results *in vivo*, we employed an orthotopic tongue xenograft mouse model using HSC3 cells. Pharmacologic blockade of CXCR1/2 with reparixin significantly reduced CXCL8 and MMP1 expression in tumor tissues, as confirmed by IHC (Figure 8J-K), but did not affect primary tumor proliferation (Figure 8I). In contrast, reparixin treatment led to a notable decrease in proliferative tumor cells and CXCL8/MMP1 levels in cervical lymph nodes (Figure 8L-N), suggesting that this signaling axis plays a more prominent role in regional metastasis rather than primary tumor growth. These findings suggest that CXCL8 contributes more prominently to regional metastasis rather than primary tumor growth. These *in vivo* data reinforce the functional role of the CXCL8-CXCR1/2 axis in OSCC metastasis and support the therapeutic potential of targeting this pathway. Interestingly, pharmacological inhibition of CXCR1/2 with reparixin in our OSCC xenograft model led to reduced CXCL8 expression in tumor tissues, suggesting the existence of a positive-feedback loop in which downstream components of the CXCL8-JAK1/STAT3 axis help to sustain CXCL8 transcription. Similar STAT3-cytokine feedback circuits have been reported in breast cancer, HNSCC, and glioblastoma 49-51. This observation raises the intriguing possibility that activated STAT3 may further enhance CXCL8 gene expression, thereby sustaining a self-reinforcing pro-metastatic signaling cycle. Similar feedback mechanisms between STAT3 and IL-8/CXCL8 have been documented in glioblastoma, HNSCC, and breast cancer, where STAT3 directly binds to the CXCL8 promoter and drives its transcription, creating a self-amplifying inflammatory loop that promotes tumor progression. In the context of OSCC, such a feedback mechanism would have important therapeutic implications, as it suggests that targeting any component of the CXCL8-CXCR1/2-JAK1-STAT3-MMP1 axis may disrupt multiple interconnected pro-metastatic processes simultaneously. Supporting this notion, our single-cell RNA-seq analysis of OSCC tissues (GSE103322) revealed that malignant epithelial cells are the predominant source of CXCL8 and co-express MMP1 (Supplementary Figure 1A-C). This indicates that tumor cells may establish an autocrine circuit, producing and responding to CXCL8 to reinforce their own metastatic capacity through STAT3 activation and MMP1 upregulation. Furthermore, to explore environmental factors that may initiate or amplify this signaling cascade, we treated HSC3 cells with arecoline, a known betel nut-derived carcinogen. Arecoline induced CXCL8 expression in a dose-dependent manner (Supplementary Figure 2), suggesting that environmental exposure could trigger or exacerbate CXCL8-driven metastasis in OSCC. This finding provides additional translational relevance, highlighting the potential of targeting the CXCL8 axis in patients with known risk exposures.

Taken together, this study provides the first comprehensive evidence that CXCL8 orchestrates OSCC metastasis by initiating a CXCR1/2-JAK1-STAT3 signaling cascade that drives MMP1 transcription. Our integration of molecular, cellular, and *in vivo* models reveals that this axis not only mediates tumor dissemination but may also be self-reinforcing. These findings position CXCL8 as both a prognostic biomarker and a therapeutic target for preventing OSCC metastasis.

## Conclusion

Our study identifies CXCL8 as a central regulator of OSCC metastasis, acting through a CXCR1/2-JAK1-STAT3-MMP1 signaling pathway. Targeting this axis effectively attenuated metastatic behavior *in vitro* and in an orthotopic xenograft model, while scRNA-seq analysis confirmed tumor cells as the primary source of CXCL8. These findings provide both mechanistic and translational insights, supporting CXCL8 and its downstream effectors as promising targets for anti-metastatic therapies in OSCC.

## Supplementary Material

Supplementary figures.

## Data Availability

The datasets generated for this study can be accessed upon request to the corresponding author.

## References

[1] Reichal P, Prethipa R (2024). A Comprehensive Retrospective Institutional Study for Decoding Oral Squamous Cell Carcinoma. Cureus.

[2] Bray F, Laversanne M, Sung H, Ferlay J, Siegel RL, Soerjomataram I (2024). Global cancer statistics 2022: GLOBOCAN estimates of incidence and mortality worldwide for 36 cancers in 185 countries. CA Cancer J Clin.

[3] Chou CW, Lin CR, Chung YT, Tang CS (2023). Epidemiology of Oral Cancer in Taiwan: A Population-Based Cancer Registry Study. Cancers (Basel).

[4] Liu JC, Bhayani M, Kuchta K, Galloway T, Fundakowski C (2019). Patterns of distant metastasis in head and neck cancer at presentation: Implications for initial evaluation. Oral Oncol.

[5] Cao M, Shi E, Wang H, Mao L, Wu Q, Li X (2022). Personalized Targeted Therapeutic Strategies against Oral Squamous Cell Carcinoma. An Evidence-Based Review of Literature. Int J Nanomedicine.

[6] Alazzawi W, Shahsavari Z, Babaei H, Firouzpour H, Karimi A, Goudarzi A (2022). The evaluation of serum lipid profile and apolipoprotein C-1 in the Iranian patients of Oral Squamous Cell Carcinoma. Biomedicine (Taipei).

[7] Ha H, Debnath B, Neamati N (2017). Role of the CXCL8-CXCR1/2 Axis in Cancer and Inflammatory Diseases. Theranostics.

[8] Song N, Cui K, Zeng L, Li M, Fan Y, Shi P (2024). Advance in the role of chemokines/chemokine receptors in carcinogenesis: Focus on pancreatic cancer. Eur J Pharmacol.

[9] Sarode GS, Sarode SC, Patil A, Anand R, Patil SG, Rao RS (2015). Inflammation and Oral Cancer: An Update Review on Targeted Therapies. J Contemp Dent Pract.

[10] Luppi F, Longo AM, de Boer WI, Rabe KF, Hiemstra PS (2007). Interleukin-8 stimulates cell proliferation in non-small cell lung cancer through epidermal growth factor receptor transactivation. Lung Cancer.

[11] Liu Q, Li A, Tian Y, Wu JD, Liu Y, Li T (2016). The CXCL8-CXCR1/2 pathways in cancer. Cytokine Growth Factor Rev.

[12] Yung MM, Tang HW, Cai PC, Leung TH, Ngu SF, Chan KK (2018). GRO-alpha and IL-8 enhance ovarian cancer metastatic potential via the CXCR2-mediated TAK1/NFkappaB signaling cascade. Theranostics.

[13] Waugh DJ, Wilson C (2008). The interleukin-8 pathway in cancer. Clin Cancer Res.

[14] Li XP, Yang XY, Biskup E, Zhou J, Li HL, Wu YF (2015). Co-expression of CXCL8 and HIF-1alpha is associated with metastasis and poor prognosis in hepatocellular carcinoma. Oncotarget.

[15] Khijmatgar S, Yong J, Rubsamen N, Lorusso F, Rai P, Cenzato N (2024). Salivary biomarkers for early detection of oral squamous cell carcinoma (OSCC) and head/neck squamous cell carcinoma (HNSCC): A systematic review and network meta-analysis. Jpn Dent Sci Rev.

[16] Landi MT, Dracheva T, Rotunno M, Figueroa JD, Liu H, Dasgupta A (2008). Gene expression signature of cigarette smoking and its role in lung adenocarcinoma development and survival. PLoS One.

[17] Lu TP, Hsiao CK, Lai LC, Tsai MH, Hsu CP, Lee JM (2015). Identification of regulatory SNPs associated with genetic modifications in lung adenocarcinoma. BMC Res Notes.

[18] Sanchez-Palencia A, Gomez-Morales M, Gomez-Capilla JA, Pedraza V, Boyero L, Rosell R (2011). Gene expression profiling reveals novel biomarkers in nonsmall cell lung cancer. Int J Cancer.

[19] Barrett T, Wilhite SE, Ledoux P, Evangelista C, Kim IF, Tomashevsky M (2013). NCBI GEO: archive for functional genomics data sets-update. Nucleic Acids Res.

[20] Heberle H, Meirelles GV, da Silva FR, Telles GP, Minghim R (2015). InteractiVenn: a web-based tool for the analysis of sets through Venn diagrams. BMC Bioinformatics.

[21] Szklarczyk D, Franceschini A, Kuhn M, Simonovic M, Roth A, Minguez P (2011). The STRING database in 2011: functional interaction networks of proteins, globally integrated and scored. Nucleic Acids Res.

[22] Szklarczyk D, Kirsch R, Koutrouli M, Nastou K, Mehryary F, Hachilif R (2023). The STRING database in 2023: protein-protein association networks and functional enrichment analyses for any sequenced genome of interest. Nucleic Acids Res.

[23] Shannon P, Markiel A, Ozier O, Baliga NS, Wang JT, Ramage D (2003). Cytoscape: a software environment for integrated models of biomolecular interaction networks. Genome Res.

[24] Chin CH, Chen SH, Wu HH, Ho CW, Ko MT, Lin CY (2014). cytoHubba: identifying hub objects and sub-networks from complex interactome. BMC Syst Biol.

[25] Shen CJ, Kuo YL, Chen CC, Chen MJ, Cheng YM (2017). MMP1 expression is activated by Slug and enhances multi-drug resistance (MDR) in breast cancer. PLoS One.

[26] Visse R, Nagase H (2003). Matrix metalloproteinases and tissue inhibitors of metalloproteinases: structure, function, and biochemistry. Circ Res.

[27] Lukaszewicz-Zajac M, Mroczko B, Szmitkowski M (2011). Gastric cancer - The role of matrix metalloproteinases in tumor progression. Clin Chim Acta.

[28] Rosenthal EL, Zhang W, Talbert M, Raisch KP, Peters GE (2005). Extracellular matrix metalloprotease inducer-expressing head and neck squamous cell carcinoma cells promote fibroblast-mediated type I collagen degradation *in vitro*. Mol Cancer Res.

[29] Mishev G, Deliverska E, Hlushchuk R, Velinov N, Aebersold D, Weinstein F (2014). Prognostic value of matrix metalloproteinases in oral squamous cell carcinoma. Biotechnol Biotechnol Equip.

[30] Li S, Pritchard DM, Yu LG (2022). Regulation and Function of Matrix Metalloproteinase-13 in Cancer Progression and Metastasis. Cancers (Basel).

[31] Xiao M, Song H, You Y, Liu M, Yang X, Wang Y (2021). Metastasis of oral squamous cell carcinoma to the parotid lymph nodes. Int J Oral Maxillofac Surg.

[32] Tang H, Hu F, Li X, Song H (2021). Analysis of influencing factors and prognosis of early postoperative recurrence, secondary tumor and metastasis of oral squamous cell carcinoma. Cell Mol Biol (Noisy-le-grand).

[33] Matsushima K, Yang D, Oppenheim JJ (2022). Interleukin-8: An evolving chemokine. Cytokine.

[34] Ghoneim HM, Maher S, Abdel-Aty A, Saad A, Kazem A, Demian SR (2009). Tumor-derived CCL-2 and CXCL-8 as possible prognostic markers of breast cancer: correlation with estrogen and progestrone receptor phenotyping. Egypt J Immunol.

[35] Sankpal NV, Fleming TP, Gillanders WE (2013). EpCAM modulates NF-kappaB signaling and interleukin-8 expression in breast cancer. Mol Cancer Res.

[36] Wang YH, Huang JH, Tian ZF, Zhou YF, Yang J (2019). The role of CXC cytokines as biomarkers and potential targets in hepatocellular carcinoma. Math Biosci Eng.

[37] Shao Y, Lan Y, Chai X, Gao S, Zheng J, Huang R (2023). CXCL8 induces M2 macrophage polarization and inhibits CD8(+) T cell infiltration to generate an immunosuppressive microenvironment in colorectal cancer. FASEB J.

[38] Paczek S, Lukaszewicz-Zajac M, Gryko M, Mroczko P, Kulczynska-Przybik A, Mroczko B (2020). CXCL-8 in Preoperative Colorectal Cancer Patients: Significance for Diagnosis and Cancer Progression. Int J Mol Sci.

[39] Li H, Nord EP (2009). IL-8 amplifies CD40/CD154-mediated ICAM-1 production via the CXCR-1 receptor and p38-MAPK pathway in human renal proximal tubule cells. Am J Physiol Renal Physiol.

[40] Hu X, Yuan L, Ma T (2020). Mechanisms of JAK-STAT signaling pathway mediated by CXCL8 gene silencing on epithelial-mesenchymal transition of human cutaneous melanoma cells. Oncol Lett.

[41] Zhang JY, Du Y, Gong LP, Shao YT, Wen JY, Sun LP (2022). EBV-Induced CXCL8 Upregulation Promotes Vasculogenic Mimicry in Gastric Carcinoma via NF-kappaB Signaling. Front Cell Infect Microbiol.

[42] Fu X, Wang Q, Du H, Hao H (2023). CXCL8 and the peritoneal metastasis of ovarian and gastric cancer. Front Immunol.

[43] Hong D, Kurzrock R, Kim Y, Woessner R, Younes A, Nemunaitis J (2015). AZD9150, a next-generation antisense oligonucleotide inhibitor of STAT3 with early evidence of clinical activity in lymphoma and lung cancer. Sci Transl Med.

[44] Goldstein LJ, Perez RP, Yardley D, Han LK, Reuben JM, Gao H (2020). A window-of-opportunity trial of the CXCR1/2 inhibitor reparixin in operable HER-2-negative breast cancer. Breast Cancer Res.

[45] Tanis T, Cincin ZB, Gokcen-Rohlig B, Bireller ES, Ulusan M, Tanyel CR (2014). The role of components of the extracellular matrix and inflammation on oral squamous cell carcinoma metastasis. Arch Oral Biol.

[46] Wu K, Mao YY, Han NN, Wu H, Zhang S (2021). PLAU1 Facilitated Proliferation, Invasion, and Metastasis via Interaction With MMP1 in Head and Neck Squamous Carcinoma. Front Oncol.

[47] Fan HX, Chen Y, Ni BX, Wang S, Sun M, Chen D (2015). Expression of MMP-1/PAR-1 and patterns of invasion in oral squamous cell carcinoma as potential prognostic markers. Onco Targets Ther.

[48] George A, Ranganathan K, Rao UK (2010). Expression of MMP-1 in histopathological different grades of oral squamous cell carcinoma and in normal buccal mucosa - an immunohistochemical study. Cancer Biomark.

[49] de la Iglesia N, Konopka G, Lim KL, Nutt CL, Bromberg JF, Frank DA (2008). Deregulation of a STAT3-interleukin 8 signaling pathway promotes human glioblastoma cell proliferation and invasiveness. J Neurosci.

[50] Xu Q, Ma H, Chang H, Feng Z, Zhang C, Yang X (2020). The interaction of interleukin-8 and PTEN inactivation promotes the malignant progression of head and neck squamous cell carcinoma via the STAT3 pathway. Cell Death Dis.

[51] Uddin N, Kim RK, Yoo KC, Kim YH, Cui YH, Kim IG (2015). Persistent activation of STAT3 by PIM2-driven positive feedback loop for epithelial-mesenchymal transition in breast cancer. Cancer Sci.

