# Supplementary material for: CXCL8 Drives MMP1 Upregulation and Promotes Metastatic Progression in Oral Cancer Through CXCR1/2-Mediated JAK1/STAT3 Activation
Source: Int J Biol Sci. 2026 Feb 1;22(5):2285–301. doi: 10.7150/ijbs.115990 (PMC12965068; doi:10.7150/ijbs.115990)
Supplement: Supplementary file 1 — Supplementary figures. [file ijbsv22p2285s1.pdf]

Supplementary Figure 1

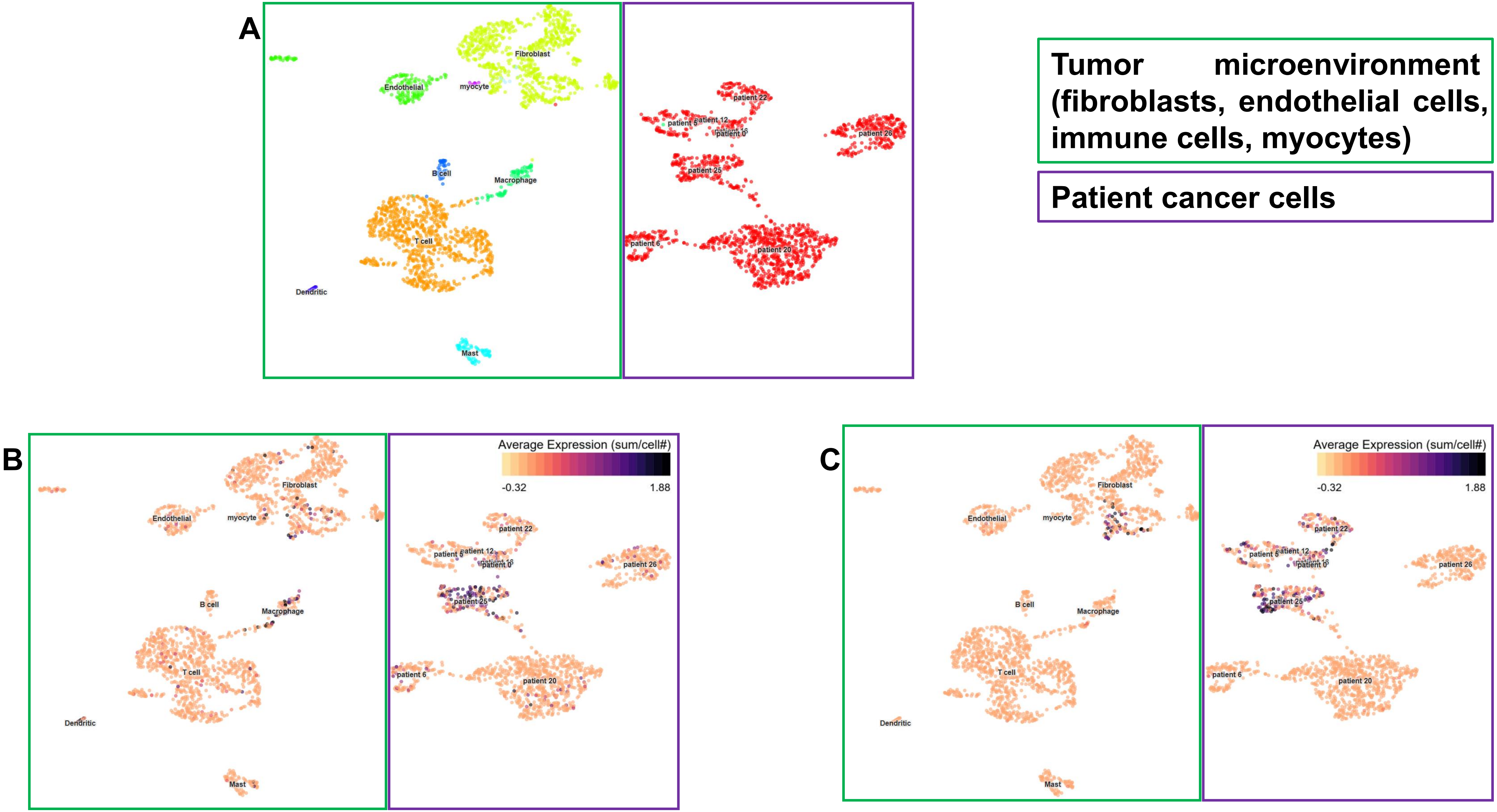

Supplementary Figure 1. Single-cell transcriptomic analysis of CXCL8 and MMP1 expression in head and neck cancer tissue. (A) Cell-type distribution and annotation of cancer cells, stromal cells, and immune cells from head and neck squamous cell carcinoma (HNSCC) patient tumors, visualized using UMAP plots via the UCSC Cell Browser platform (GSE103322). (B) CXCL8 gene expression levels in different cell populations within the tumor microenvironment.(C) MMP1 gene expression levels in corresponding cell types. Data were retrieved and visualized from public single-cell RNA-seq dataset (GSE103322) using the UCSC Cell Browser platform.

Supplementary Figure 2

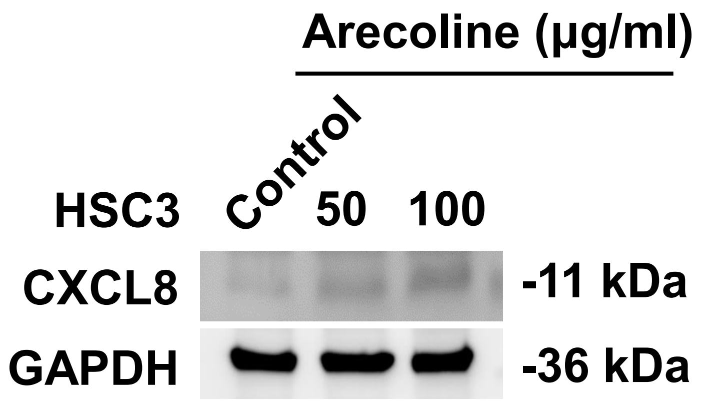

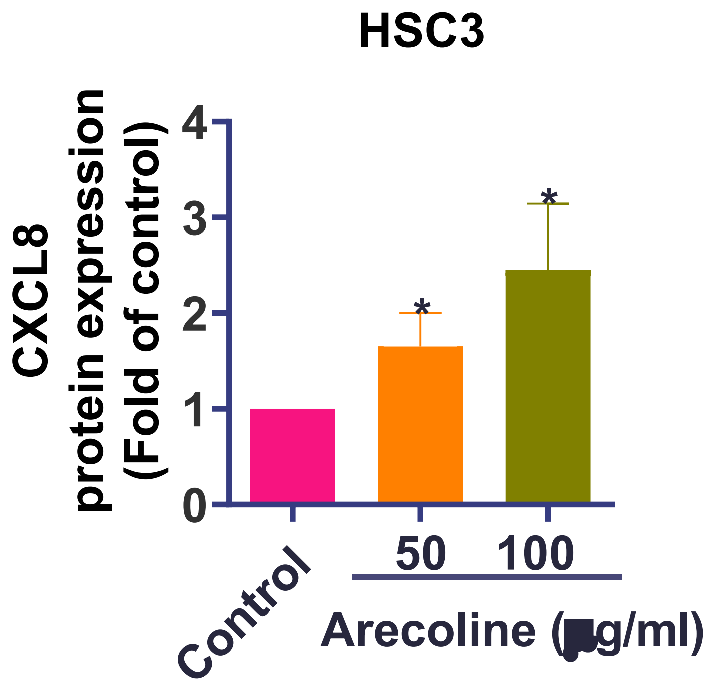

Supplementary Figure 2. Arecoline induces CXCL8 protein expression in oral cancer cells. Western blot analysis of CXCL8 protein levels in HSC3 cells treated with increasing concentrations of arecoline (0, 50, and 100 μg/mL) for 24 hours. GAPDH was used as a loading control (n=4). Results are expressed as the mean ± SD of four independent experiments. $p < 0.05$ was considered statistically significant.
